# Improving Evidenced-based Outpatient Order Set Utilization in the Gastrointestinal Division of a Large Pediatric Health System

**DOI:** 10.1097/pq9.0000000000000792

**Published:** 2025-01-23

**Authors:** Kevin L. Watson, April M. Love, Hanna Lemerman, Cathy Gustaevel, Prabi Rajbhandari

**Affiliations:** From the * Division of Gastroenterology, Hepatology and Nutrition, Akron Children’s Hospital, Akron, Ohio; † Quality Services Department, Akron Children’s Hospital, Akron, Ohio; ‡ Division of Primary Care, Akron Children’s Hospital, Akron, Ohio; § Division of Hospital Medicine, Akron Children’s Hospital, Akron, Ohio.

## Abstract

**Introduction:**

Standardization is crucial in improving healthcare outcomes, equity and quality. Clinical decision support tools are key to achieving this goal. At our organization, Epic serves as our electronic health record, and SmartSets are Epic’s version of outpatient standardized order sets with embedded clinical decision support tools. In 2022, the utilization of SmartSets in our hospital’s gastrointestinal division was only 1.9%, far below our organizational target of 50%.

**Methods:**

Our group formed a quality improvement (QI) team and chose the model for improvement methodology. The interventions focused on education, buy-in, feedback performance monitoring, and the enhancement and development of new SmartSets. Our primary aim was to increase the utilization rate of SmartSets by gastrointestinal providers from 1.9% to 20%, and our secondary aim was to reduce the time spent by providers on orders by 10% from 3.3 to 2.8 minutes per encounter. Our balancing measure was monitoring safety reports during the study period.

**Results:**

SmartSet utilization improved to greater than 20% within 7 months of the project initiation. Three months after implementing SmartSet updates and introducing new SmartSets into production, time spent on orders during clinical encounters decreased from a median of 3.3 to 2.4 minutes per encounter. We appreciated that there was no change in safety reporting during the project timeline.

**Conclusions:**

We achieved our goal of improving utilization rates of standardized SmartSets and reducing time spent on orders using a QI methodology. Our achievements underscore the effectiveness of QI methods in enhancing SmartSet utilization and streamlining order processes.

## INTRODUCTION

Divergent clinical practices among providers in a health system are known to cause harmful effects on patient care, including medication errors and increased safety events.^1,2^ Standardization of practices can help to alleviate these concerns through improved adherence to clinical guidelines through various measures.^1,3^ The advent of electronic health records (EHRs) has helped to make strides in improving standardization with utilities such as clinical decision support (CDS) systems.^3–5^ CDS systems have a range of tools to improve decision-making within the clinical workflow, and they have been in place for decades to assist healthcare providers.^2,3,6^ They are critical in various areas of healthcare in improving overall care and outcomes.^5,7,8^ Standardized order sets are a form of CDS that help clinicians prescribe appropriate medications with correct dosages and proper laboratory testing based on disease-specific guidelines.^9,10^ They are typically synthesized with features such as computerized physician order entry (CPOE), which reduces errors, improves guideline adherence and decreases costs.^7,11,12^ For these reasons, investigators previously performed various studies and QI projects to evaluate how to improve the utilization of standardized order sets in various fields of medicine.^12–15^

Providers can use order sets containing CPOE and CDS in various clinical settings within an EHR. Standardized order sets can be enhanced through education, EHR modification, guidelines, literature reviews, and subject matter expert involvement for optimal performance and efficiency.^4,16,17^ Standardizations such as these have previously demonstrated a decreased burden on outpatient healthcare providers and have a beneficial impact on provider burnout.^1,3,15,18^ At our organization, Epic (Epic Systems, Verona, Wis.) is our EHR.^19^ SmartSets are Epic’s version of outpatient standardized order sets with embedded CPOE, CDS, and other specialty-specific information to guide healthcare providers in appropriate and timely patient management.^11,15^ These SmartSets have been widely used throughout primary care practices in our pediatric health system. However, our hospital’s Smart Set usage rate by specialty divisions has historically been low. Other organizations have demonstrated similar difficulties in standardized order set utilization in various specialties, including gastrointestinal (GI) divisions.^20–22^

Our organization has set a goal to utilize outpatient SmartSets in at least 50% of outpatient clinical encounters. During the last 6 months of 2022, utilization of SmartSets within the GI division was at 1.9%, leaving much room for improvement. We initiated a quality improvement (QI) project to enhance the utilization of SmartSets and to align it with the hospital’s organizational objectives. Our primary aim was to increase SmartSet utilization by the GI division at our institution from 1.9% to 20% by June 23, 2023. Our secondary aim was to decrease the time spent on orders during outpatient clinical encounters by GI providers by 10% from a baseline median of 3.3 to 2.8 minutes by the end of the study period.

## METHODS

This QI project was conducted at a freestanding academic children’s hospital and healthcare system in the Midwest, serving more than 1 million outpatient clinic visits annually. The GI division comprises 15 healthcare providers, including 9 physicians and 6 nurse practitioners. They offer care to patients between 0 and 25 years of age at 10 different regional locations within the healthcare system. The healthcare providers in this division conduct more than 20,000 outpatient clinic visits annually. The GI physicians provide inpatient and outpatient services, and inpatient service is rotated equally among the physicians who take 1 week of inpatient service at a time. The GI physicians typically do not provide outpatient clinical services during their inpatient service week. The nurse practitioners only provide outpatient clinic services. The intervention period was 9 months from February to October 2023, with a baseline period of January 2023 and a postintervention period of 2 months from November 2023 to December 2023.

We assembled a multidisciplinary QI team consisting of a clinical care standardization specialist, a quality initiative specialist, an Epic SmartSet committee leader, a GI division quality liaison and a GI physician serving as project leader. The Institute for Healthcare Improvement Model for Improvement was used for the improvement methodology.^23^ The institutional review board reviewed the QI project and deemed it exempt from oversight.

### Interventions

#### Education and Obtaining Buy-in

**Fig. 1.**
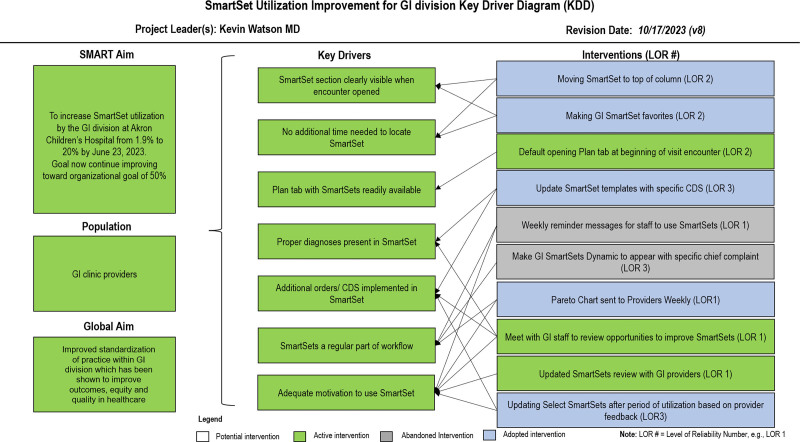
Key driver diagram detailing interventions and goals for the SmartSet utilization project.

One of our institution’s primary goals was to improve care standardization, and increasing SmartSet utilization is one way the institution hopes to achieve this goal. By aligning this project with previously set institutional goals, informatics and information technology leadership buy-in and support to improve SmartSet utilization were easily obtained. After an initial discussion with the QI team, we conducted a system failure mode analysis to assess processes and potential interventions. We created a key driver diagram (Fig. 1) and designed interventions to target the key drivers. We presented the project’s aims and goals to other members of the GI division, including how the QI project aligned with institutional needs. We requested feedback from the GI team regarding their workflow and what features may make SmartSets more likely to be utilized.

#### Promoting Accessibility

In the previous standard EHR setup, the SmartSet panel appeared at the bottom of the screen, requiring providers to scroll down during clinic workflow to access it. After scrolling to the bottom of the screen, they had to initiate a search prompt to find the SmartSet to access it. This requirement hindered using SmartSets as they were not easily accessible. We initiated 2 plan-do-study-act (PDSA) cycles at the beginning of the project, aiming to make SmartSets more visible. These interventions included (1) adjusting the location of SmartSets, so they appear at the top of the screen and are more aligned with the workflow and (2) teaching to save GI SmartSets as favorites to eliminate additional searching. Initially, only 2 GI providers were given instructions for implementing these changes. Following implementation, these providers reported improved workflow and increased likelihood of SmartSet usage. Subsequently, we iteratively continued these interventions as we included additional GI providers.

#### Continued Education, Feedback, and Performance Monitoring

To improve motivation for using SmartSets, we initiated additional PDSA cycles, including (1) sending reminder messages to providers about utilizing SmartSets and (2) sending weekly Pareto charts on SmartSet usage to GI providers. Reminder messages were sent weekly via text by the QI project leader to GI providers. Later in the project, we sent reminder messages twice a week. Despite these efforts, there were no significant changes in the utilization rate. This process was also not sustainable in the long term. Hence, we discontinued the intervention.

The weekly Pareto charts gave healthcare providers insights into their utilization rates and allowed them to compare their performance. Initially, the Pareto charts were anonymous, and providers could inquire about their status. In the subsequent PDSA cycle, we removed anonymity based on providers’ feedback that it brought out a “competitive nature” and motivated certain providers to improve their usage.

#### Enhancing Available SmartSets

Throughout the above PDSA cycles, GI providers were encouraged to provide input regarding SmartSets. Initially, 12 GI SmartSets were in production; however, these SmartSets lacked disease-specific modifications and system enhancements as the GI group had not updated them in several years. After receiving feedback, we incorporated CDS tools such as clinical guidelines, hyperlinks, patient education and clinical support algorithms into the SmartSets. This process of integrating feedback was swift and efficient, ensuring that the SmartSets were constantly evolving to meet the needs of our providers. The specific adjustments made in each SmartSet are listed in Table 1. All 12 GI SmartSets were enhanced and updated during this process. In the future, we will make different SmartSets for reflux for various ages of development.

**Table 1.** All Updated GI SmartSets Listed with Modifications and Enhancements Made, Divided into Enhancement Categories

Name of SmartSet	Medications	laboratory/Imaging	CDS	Education
Abdominal pain	Separate categories of medication based on type New category for antispasmodics and chronic IBS-C medications added	No changes	Dosing recommendations for new IBS-C medications	Patient education for IBS and abdominal pain added
Celiac disease	Antispasmodic medications added	Additional laboratory results added	Hyperlink added to celiac disease decision tree tool available on the web Laboratory guidance for yearly celiac disease monitoring	Patient education added on celiac disease, gluten free diet and nonceliac gluten sensitivity
Constipation	New chronic IBS-C medications added Medications divided by type (ie, osmotic laxatives, stimulant laxatives, lubricants)	Additional laboratory results added	Dosing recommendations for new IBS-C medications	Patient education added for fiber and fluid supplementation based on age New patient education instructions for bowel cleanout
Diarrhea	Medications for IBS-D added as well as potential antibiotics	Additional laboratory results added Stool studies updated based on current hospital availability and preferences	No changes	Patient instructions for collecting stool sample added Patient education added for diarrhea, dehydration and toddler’s diarrhea
EoE	New medications added Medications divided by category	No changes	Guidance on prescribing new EoE medications	Patient education on eosinophilic esophagitis
Failure to thrive	No changes	Labs divided by category and stool testing added	Updated failure to thrive guidelines added	Patient education on growth delay or disorder
Feeding problems	Thickening agents and formulas added	No changes (Swallow study panel already present)	No changes	Patient education on gastrostomy care and picky eaters
IBD	Medications divided by category and new IBD medications added	Laboratory results divided by category	Laboratory monitoring instructions added as well as health maintenance	Patient education for IBD added and patient instructions for health maintenance
Liver disease	Medications divided by category and new hepatitis C medications added	Laboratory results divided by category New available imaging added	Laboratory monitoring instructions added for various liver disease Hyperlinks to published guidelines added	Patient information added for various liver disease
Pancreatitis	Pancreatic enzymes added. Medication divided into categories	Additional labs for monitoring chronic pancreatitis added	Updated pediatric pancreatitis guidelines added	Patient education for chronic and acute pancreatitis added
Reflux	No changes (thickening agents and formulas already present)	Swallow study panel added (upper GI study already present)	Link to updated reflux guidelines	Patient education added on Barrett’s esophagus and spitting up infant
Short bowel syndrome	Medications divided into categories	Additional laboratory results added based on guidelines. Laboratory results subdivided into categories	Guidance on monitoring patients on parenteral nutrition	Patient education on parenteral nutrition added

EoE, eosinophilic esophagitis; IBS-C, irritable bowel syndrome with constipation; IBS-D, irritable bowel syndrome with diarrhea.

#### Additional SmartSet Development

**Fig. 2.**
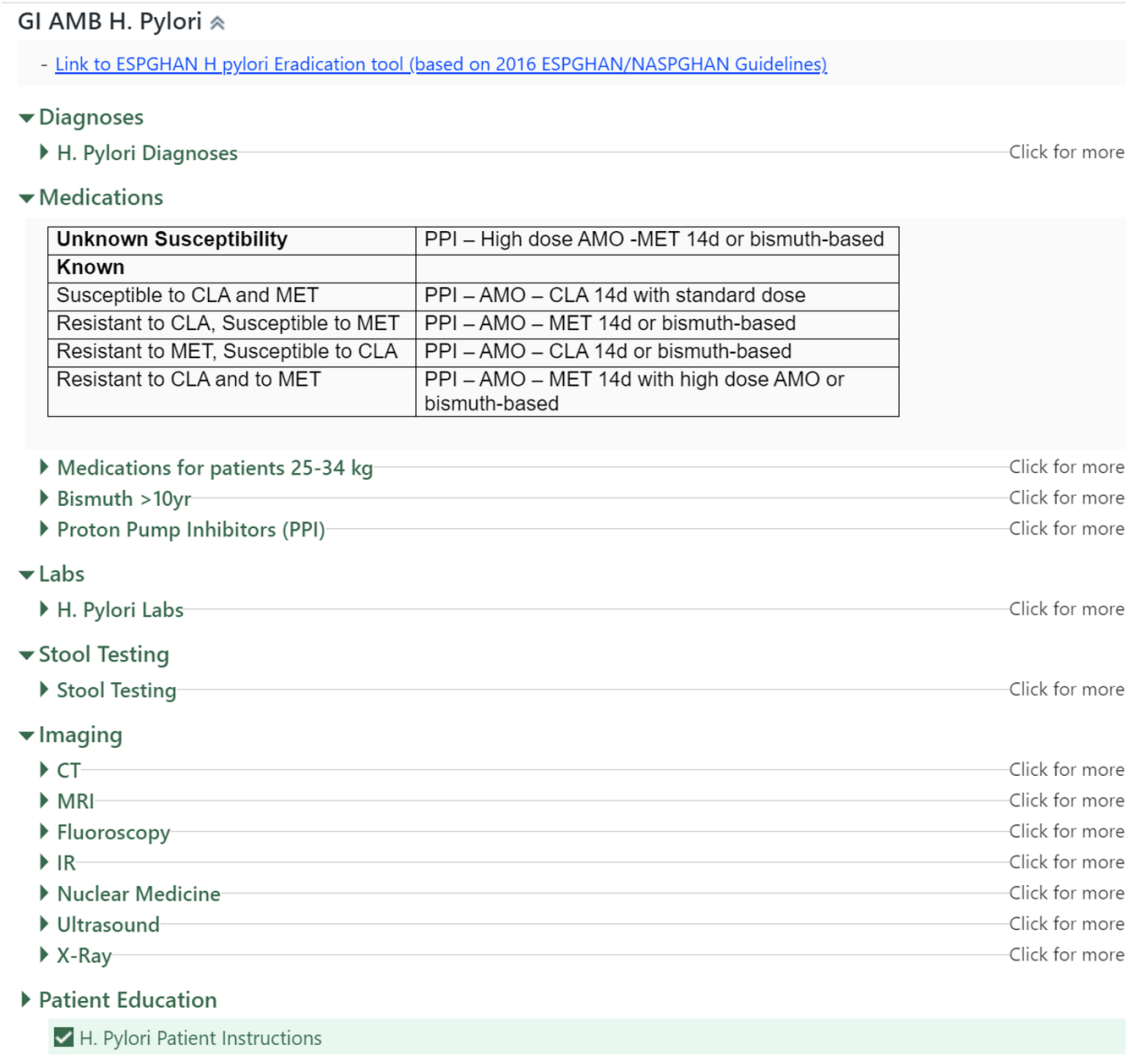
Example of newly developed GI SmartSet for *H. pylori*. It includes hyperlinks to the most recent available treatment guidelines, and antibiotic treatment recommendations based on susceptibilities and has all potential antibiotic choices available as drop down. Medication category groups are dynamic based on patient age and weight and certain choices only appear if patients meet rules set within the system for this SmartSet. Patient education regarding *H. pylori* is also prechecked and will be distributed to the patient’s family upon discharge. *H. pylori*, Helicobacter pylori.

At the onset of our QI project, our GI division had 12 available SmartSets. Recognizing the need for more comprehensive tools, we expanded the range of SmartSets within our division, which took approximately two months from initiation to implementation. During this process, we created a total of three SmartSets. Figure 2 demonstrates one of the new GI SmartSets created with details on implemented enhancements. Before integrating the new SmartSets into our workflow, we convened with all GI providers and obtained their feedback. We considered all feedback and made adjustments. The SmartSets were thus optimized. This collaborative approach ensured the SmartSets met our providers’ specific needs and preferences. Approximately one month after deploying the new SmartSets, we distributed a short survey of 5 Likert-scale questions to all GI providers. Six providers responded to the survey that included questions about how often they used SmartSets, their comfort level using SmartSets, their feelings about how SmartSets improve workflow, their seeing value in SmartSets and if they felt SmartSets were a waste of time. The results indicated the majority of respondents felt they enhanced their workflow.

### Measures

#### Primary Measure

The primary measure of our study was the percentage of SmartSet utilization among GI providers. We generated these data via a weekly report from the EHR detailing SmartSet usage for each provider. Outpatient GI clinic encounters utilizing SmartSets served as the numerator, with all outpatient GI encounters as the denominator, with data further broken down per provider.

#### Secondary Measure

The secondary measure of our study was the change in time spent in “orders” during appointments for GI providers. The “orders” section is a separate area where clinicians place orders in the EHR. Using the EHR, we can accurately calculate the time spent by each provider in this section. The beginning of the order time is marked when the provider clicks to initiate orders, and the end is marked when they exit this section after completing. We generated a report from Epic Signal data during the study period and assessed monthly for all GI providers and per provider. The numerator is the minutes providers spent in an orders activity for outpatient encounters within the reporting period and the denominator of appointments within the reporting period.

#### Balancing Measure

Our balancing measure was to assess monthly safety event reporting in the GI division for medication errors, adverse events, or other patient safety incidents throughout the project period. Safety events are entered through the Midas Plus event reporting system (Molecular Dimensions, Inc., Holland, Ohio).^24^ Any employee in the organization can enter these events, which the hospital quality team monitors daily. The hospital quality team includes a multidisciplinary group of professionals, such as a director of patient safety, Midas coordinator, QI specialists, and clinical leaders, including physicians and nurse managers, who provide clinical expertise. This team reviews these events, investigates them, and relays information to the necessary persons and departments. They will also determine if further apparent cause analysis or root cause analysis is needed. We tracked safety event reports from outpatient GI appointments throughout the QI project timeline to ensure there was no increased reporting of safety events.

We used a p-chart for the primary measure, and for the secondary measure, we used an x-chart to assess the impact of improvement efforts for the primary and secondary aims. These are statistical process control charts using the binomial distribution. A run of ≥8 points in a row above or below the centerline was considered a shift.

## RESULTS

### Primary Measure

**Fig. 3.**
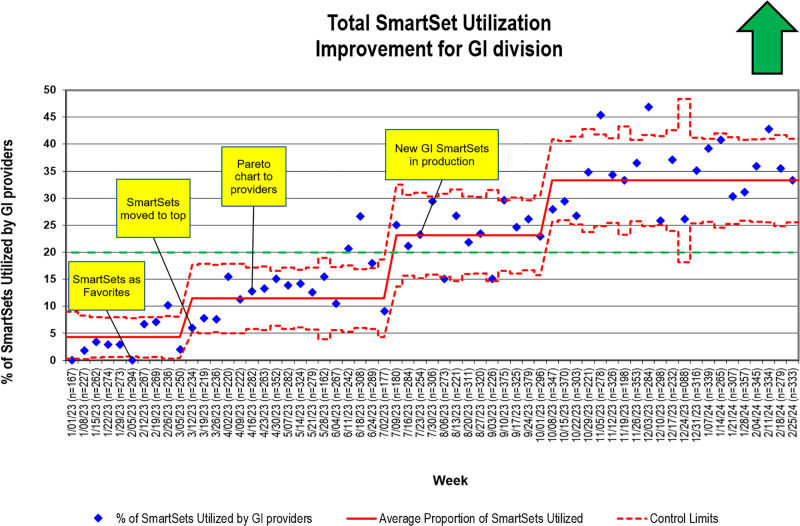
Control chart for SmartSet improvement utilization project.

The baseline usage of SmartSets among GI providers was 1.9%. Our baseline period was 5 weeks between January 1, 2023, and Febreuary 5, 2023. We made our first shift 3 months into the project, and our usage increased to 6.7%. This shift occurred after introducing the project and education for favoriting of SmartSets. We observed 2 subsequent shifts indicating increasing usage, and we had our first data shift above our goal of 20% in August 2023 (Fig. 3). We have sustained the SmartSet utilization for 4 months since project completion on December 31, 2023. We reviewed post hoc SmartSet data for 2 months following project completion, with the sustainment of previous goals noted as well as the final data shift.

### Secondary Measure

**Fig. 4.**
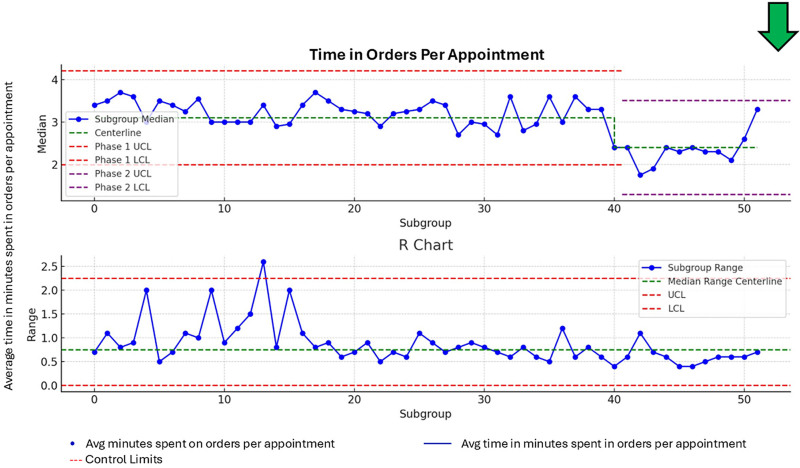
Control chart for time spent in orders for outpatient clinic appointments.

The baseline median time on orders per provider during outpatient clinical encounters was 3.3 minutes. Twelve months into the project, the average time spent per provider in the orders section improved to 2.4 minutes (Fig. 4).

### Balancing Measure

During 2023, 30 potential safety events related to the GI division were entered. Of those, 19 were related to outpatient clinical services within GI. Eleven of these events occurred before the new and updated SmartSets went into production, and 8 occurred after their implementation in July 2023. Nine events were categorized as test/procedure/treatment complication or delay, 5 were classified as missing/incorrect medication or formula, and the remainder were secondary to patient information or privacy concerns. We performed further in-depth exploration by assessing the hospital quality team report of events, including independent assessment of those involved, including the GI management team and any staff and providers in any of these named incidents, to see if any of these events could be associated with SmartSet usage. The independent review by the hospital quality team did not demonstrate any correlation between the events and SmartSet CDS tool usage.

## DISCUSSION

We successfully achieved the primary aim of our project by increasing the SmartSet utilization in the GI division from 1.9% to 20%. Although we did not meet our initial timeline goal of June 2023, we did meet our goal within 7 months of study initiation, and we have sustained the improvements. Following QI principles, we initially set a 20% utilization target to support gradual and sustainable adoption within 7 months. Still, we were uncertain how GI providers would respond to the change in workflow. Despite aiming for a lower threshold, we demonstrated more than 30% improvement in utilization and plan to use these findings for more ambitious targets in future phases. Additionally, we effectively reduced the provider’s median time spent in the orders section of the EHR during outpatient clinical encounters from 3.3 to 2.4 minutes per encounter, representing a 25% decrease in time. These findings suggest that increased usage of standardized and critically reviewed order sets, such as SmartSets, can significantly decrease the time spent ordering during outpatient clinical encounters.

During our project, we noticed that the most effective approach was obtaining provider buy-in and getting their input in designing the CDS. This collaborative effort ensured we tailored the SmartSets to meet our providers’ specific needs and workflows, leading to greater acceptance and utilization. This observation highlights the importance of engaging frontline staff in developing and implementing QI initiatives to maximize their impact and sustainability. We also noticed several episodes of special cause variation as indicated by values outside the chart’s control limit. One potential explanation was that the temporary surge in the use of SmartSets was due to the initial influence of these interventions, hence an observable increase in the adoption of SmartSets by providers. However, over time, providers returned to their usual or previous patterns of behavior, leading to a decline in the use of SmartSets back to levels that are within control limits or typical for the setting. Another contributing factor is likely the scheduling of providers. The GI providers provide inpatient and outpatient services. When a provider who typically uses SmartSets extensively is assigned to inpatient services, or conversely, a provider with minimal usage is on outpatient services, this variability in individual usage patterns can significantly impact overall SmartSets utilization and has the potential to introduce special cause variations in the data as there is not a consistent pattern with provider inpatient service schedules due to vacation time and other personal and professional obligations.

Previous studies have shown that improving the utilization of standardized order sets and additional CDS tools can reduce provider clinical burden by decreasing variability and the amount of time spent in the EHR during clinical encounters.^12,14^ Clinical workload reduction has been shown to improve the perception of provider burnout in previous studies.^18,25^ With increasing SmartSet usage in our study, we saw a decrease of 25% in time on orders during outpatient clinical encounters in the GI clinic. This reduction in time allows providers to allocate more time to essential patient tasks and regain much-needed time during the day. The decrease in time spent on orders in encounters within the EHR, through interventions such as those implemented in this QI project, may help to reduce cognitive load and ease burnout perception in healthcare providers; this, in turn, may contribute to improved provider well-being and enhance the quality of patient care.^26^

Our study has certain limitations. This intervention was undertaken within a GI division in an academic freestanding children’s health system with a robust dedicated EHR team, and, as such, the results may not be replicable in other healthcare settings. Similarly, although we reached our goal of increasing the utilization of SmartSets and reducing time spent by providers on orders, we did not measure how this impacts patients, families, and providers. We did not measure if the reduction in time spent on orders contributed to burnout in any way; thus, any perceived benefit is indirectly estimated based on previously mentioned results. Additionally, although we used and reviewed the safety event reporting system for potential harmful events related to the intervention, this was not designed systematically, potentially introducing bias during the review.

Overall, this QI project demonstrated significant improvement in SmartSet utilization with GI providers in our pediatric health system and a remarkable 25% decrease in order-related time during outpatient clinical encounters. Other subspecialty divisions and healthcare settings can apply these findings. By leveraging the lessons learned and interventions implemented in our study, we anticipate that other healthcare facilities can replicate and adapt similar QI initiatives to achieve comparable outcomes. This approach can optimize efficiency, streamline workflows, enhance provider satisfaction, and improve patient care across diverse healthcare environments.

## ACKNOWLEDGMENTS

The authors thank Akron Children’s Hospital’s Project-to-Publication (P2P) team for their invaluable guidance and support throughout the manuscript writing process—a special thanks to Elizabeth Smith for her assistance with our control charts.
